# Flexible, Transparent, and Cytocompatible Nanostructured Indium Tin Oxide Thin Films for Bio-optoelectronic Applications

**DOI:** 10.1021/acsami.3c10861

**Published:** 2023-09-22

**Authors:** Katarzyna Krukiewicz, Dominika Czerwińska-Główka, Roman Maria Turczyn, Agata Blacha-Grzechnik, Catalina Vallejo-Giraldo, Karol Erfurt, Anna Chrobok, Jérôme Faure-Vincent, Stéphanie Pouget, David Djurado, Manus J.P. Biggs

**Affiliations:** † Department of Physical Chemistry and Technology of Polymers, Silesian University of Technology, 44-100 Gliwice, Poland; ‡ Centre for Organic and Nanohybrid Electronics, Silesian University of Technology, 44-100 Gliwice, Poland; § Centre for Research in Medical Devices, University of Galway, H91 TK33 Galway, Ireland; ∥ Department of Chemical Organic Technology and Petrochemistry, Silesian University of Technology, 44-100 Gliwice, Poland; ⊥ CEA/INAC/SPrAM, Laboratoire d’Electronique Moléculaire Organique et Hybride, 38000 Grenoble, France

**Keywords:** bio-optoelectronics, deep brain stimulation, electrochemical modification, indium tin oxide, nanostructured ITO, neural interfaces

## Abstract

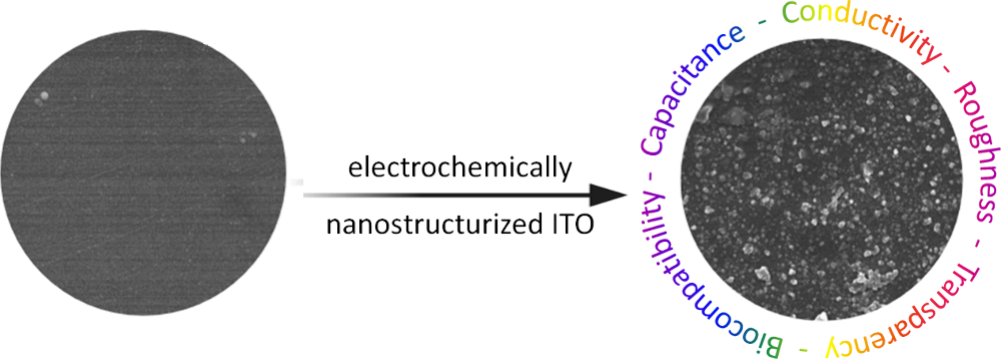

Electrical stimulation has been used successfully for several decades for the treatment of neurodegenerative disorders, including motor disorders, pain, and psychiatric disorders. These technologies typically rely on the modulation of neural activity through the focused delivery of electrical pulses. Recent research, however, has shown that electrically triggered neuromodulation can be further enhanced when coupled with optical stimulation, an approach that can benefit from the development of novel electrode materials that combine transparency with excellent electrochemical and biological performance. In this study, we describe an electrochemically modified, nanostructured indium tin oxide/poly(ethylene terephthalate) (ITO/PET) surface as a flexible, transparent, and cytocompatible electrode material. Electrochemical oxidation and reduction of ITO/PET electrodes in the presence of an ionic liquid based on d-glucopyranoside and bistriflamide units were performed, and the electrochemical behavior, conductivity, capacitance, charge transport processes, surface morphology, optical properties, and cytocompatibility were assessed in vitro. It has been shown that under selected conditions, electrochemically modified ITO/PET films remained transparent and highly conductive and were able to enhance neural cell survival and neurite outgrowth. Consequently, electrochemical modification of ITO/PET electrodes in the presence of an ionic liquid is introduced as an effective approach for tailoring the properties of ITO for advanced bio-optoelectronic applications.

## Introduction

Electrical stimulation has been used successfully for several decades for the treatment of multiple neurological disorders, including chronic pain,^1^ epilepsy,^2^ depression,^3^ and motor disorders,^4^ with hundreds of new bioelectric medicine devices currently at the clinical trial stage.^5^ In particular, deep brain stimulation (DBS) using penetrating electrode arrays has been shown to significantly reduce Parkinson’s disease-related essential tremor.^6^ A significant limitation of existing electrical neuromodulation technologies is related to the instability of the system’s impedance at the tissue/electrode interface, thought to be derived from the mechanical trauma during electrode insertion,^7^ blood vessel disruption,^8^ and the onset of reactive gliosis and electrode encapsulation.^9^ Consequently, to maintain chronic functionality of penetrating electrode systems, the stimulation signal needs to be constantly adapted, for example, by modifying the stimulation rate, stimulation amplitude, and pulse width to negate the electrical instability of the electrode/tissue interface.^10^ This instability is confounded by the need to develop increasing smaller stimulation devices, which necessitates the use of high potentials for the propagation of therapeutic alternating currents. Critically, the upper safety limit of charge injection in tissue is mediated by the potential window for water electrolysis.^11^ Consequentially, there is a pressing need to develop minimally invasive, selective, and smart electrical neuromodulation technologies capable of low-voltage neural modulation.

To this end, optogenetic approaches, which integrate optical and genetic neuromodulation technologies, have been employed to render neuron cells susceptible to stimulation via light, providing optical control of action potential generation in defined neuronal populations.^12,13^ In particular, infrared neural stimulation (INS),^14^ a technique that exploits the absorption of infrared light by the water in the tissues, can achieve a high spatial resolution^15^ and has already been employed in the peripheral^16^ and central nervous system,^17^ both in vitro^18^ and in vivo.^19^ Furthermore, recent research^20^ has demonstrated the efficacy of a dual neural stimulation using optoelectrical approaches, which employ subthreshold electrical pulses to enhance nerve excitation with high spatial precision.^21−23^ Clearly, there exists a need for new electrode materials that combine high flexibility and optical transparency necessary for optoelectronic applications with excellent electrochemical characteristics required for electrical stimulation.^24^

In particular, indium tin oxide (ITO) has been employed extensively in biomedical engineering since 1985, when Gross et al.^25^ described the fabrication of thin-film ITO electrodes for extracellular, multisite recording in neuronal cultures. Recent studies into ITO functionalization approaches have explored electrochemical methods to nanostructure ITO electrode surface for enhanced biocompatibility.^26−28^ ITO has been morphologically modified to present surface nanorods,^29^ nanowhiskers,^30^ and nanohelixes,^31^ while retaining high conductivity and transparency for the fabrication of light-emitting diodes and solar cell technologies. Due to its high surface-to-volume ratio^32^ and biomimetic structure,^33^ it is expected that nanostructured ITO should be able to promote advantageous responses at the tissue interface, facilitating cell adhesion and electrode integration, as well as enhancing recording capacity.

In a previous study by Vallejo-Giraldo et al.,^34^ an electrochemical process (anodization) was shown to enhance the electrochemical, physical, and cytocompatibility properties of ITO electrodes in vitro. On the other hand, Bouden et al.^35^ showed that the electrochemical reduction of ITO can lead to drastic morphological changes, while maintaining the electrode’s transparency. Encouraged by these results, in this study, we had investigated the effects of electrochemical oxidation and reduction on the electrochemical, topographical, and biocompatibility properties of ITO/poly(ethylene terephthalate) (PET) substrates, with an aim of fabricating modified ITO devices for bio-optoelectronic applications. To enhance the efficiency of the process and the biocompatibility of fabricated materials, the process of electrochemical nanostructurization was performed in the presence of an ionic liquid based on a d-glucopyranoside derivative as the cation precursor and a bistriflamide anion.^36^ Ionic liquids are acknowledged as environmentally friendly solvents for numerous electrochemical processes, with enhanced efficiency and without the need for harsh conditions (temperature, pressure, etc.).^37^ Although the majority of ionic liquids is known to exhibit in vitro cytotoxicity toward living organisms,^38^ it was hypothesized that sugar-derived ionic liquids can overcome these toxicity limitations and become an additional nutrient for cultured neurons, facilitating their growth and development. Here, it was shown that under selected electrochemical conditions, ITO/PET films could be topographically and chemically modified to promote the adhesion of primary neural cells and neurite outgrowth while maintaining the electrode’s transparency.

## Materials and Methods

### Electrochemical Modification of ITO/PET

Electrochemical oxidation and reduction processes were used to modify the surface of ITO/PET (Sigma-Aldrich, PET thickness: 5 mil, ITO thickness: 130 nm, surface resistivity: 60 Ω/sq, and transmittance at 550 nm >78%). For this purpose, a three-electrode electrochemical cell was equipped with an ITO/PET working electrode (0.283 cm^2^), a platinum coil auxiliary electrode, and Ag/AgCl (3 M KCl) reference electrode and connected to a PARSTAT 2273 potentiostat. The electrochemical modification was realized by the application of a constant potential of −9, −6, −3, 3, 6, and 9 V (vs Ag/AgCl) for 300 s each, in an electrolytic solution consisting of phosphate-buffered saline (PBS, Sigma-Aldrich, 0.01 M phosphate buffer, 0.0027 M KCl, and 0.137 M NaCl, pH = 7.4), 10 μM sodium poly(styrenesulfonate) (PSS, Sigma-Aldrich, *M*_W_ = 70,000 g/mol), and 0.1 M (2-d-glucopyranosyloxyethyl)trimethylammonium bistriflamide (GluIL, *M*W = 546.46 g/mol), synthesized according to the procedure described previously.^36^

### Material Characterization

Electrochemical characterization of electrochemically modified ITO/PET was performed with the use of a PARSTAT 2273 potentiostat in the same three-electrode setup as described above. Cyclic voltammetric (CV) curves were collected in the potential range from −0.5 to 0.5 V (vs Ag/AgCl) for 3 potential cycles at the scan rate of 100 mV/s in a PBS solution. Unmodified and modified ITO electrodes were also analyzed in the presence of a redox probe, K_4_[Fe(CN)_6_] (Sigma-Aldrich). CV scans were collected in 0.1 M KCl solution containing 5 mg/mL K_4_[Fe(CN)_6_] within a potential range from −0.5 to 1.0 V (vs Ag/AgCl) at a scan rate of 10 mV/s. Currents at a cathodic peak were used to calculate a relative change in an electroactive surface area (ESA) based on a Randles–Sevcik equation:^39^
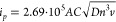
where *i*_p_ is the peak current (A), *n* is the number of electrons contributing to the redox reaction, *A* is the area of the electrode (cm^2^), *C* is the concentration of Fe(CN)_6_^4–^ in the bulk solution (mol cm^–3^), *D* is the diffusion coefficient of Fe(CN)_6_^4–^ in KCl solution (6.3 × 10^–6^ cm^2^ s^–1^^40^), and υ is the scan rate (V s^–1^).

Electrochemical impedance spectra (EIS) were collected in a PBS solution within a frequency range from 100 mHz to 10 kHz, with an AC amplitude of 40 mV (vs Ag/AgCl) and a DC potential of 0 V (vs Ag/AgCl). The results were presented as Bode plots and compared to those of an unmodified ITO/PET. EIS Spectrum Analyzer 1.0 software^41^ and the Powell algorithm were used to fit the experimental data to an equivalent circuit model. Capacitance was calculated based on the parameters of a constant phase element (CPE) as described previously.^42^ CV curves were used to determine charge storage capacity (CSC) as described previously.^43^

Transport properties of ITO/PET were evaluated with the use of a standard four-probe sensing method through the combination of a Keithley 220 current source and two Keithley 6512 electrometers. A dynamic helium flow cryostat (Oxford Instruments CF 1200 D) was used to vary the temperature from 300 to 4 K.

The transparency of ITO/PET electrodes was determined with the use of a Hewlett-Packard 8453 UV–vis diode array spectrophotometer in the wavelength range from 400 to 800 nm. The surface morphology of the samples was examined through scanning electron microscopy (SEM) with an accelerating voltage of 15 kV (Hitachi S-4700 cold field emission gun scanning electron microscope). The samples were sputter coated with a 10 nm gold layer for a better image quality for 3 min at 25 mA. Energy-dispersive spectroscopic (EDS) analysis was performed using a Phenom Pro-X scanning electron microscope, coupled with an EDS detector, operating at 15 kV. Surface roughness of samples (*S*_a_), expressed by the arithmetical mean height, was determined by means of a Profilm 3D optical profilometer.

X-ray photoelectron spectroscopy (XPS) analysis was performed with a PREVAC EA15 hemispherical electron energy analyzer with a 2D multichannel plate detector and an AlKα X-ray source (PREVAC dual-anode XR-40B source, excitation energy equal to 1486.60 eV). The base pressure was equal to 9 × 10^–9^ Pa. Survey spectra were collected at a pass energy of 200 eV (scanning step equal to 0.9 eV), which was lowered to 100 eV (scanning step equal to 0.05 eV) for high-resolution spectra. C–C component of C1s spectra (284.8 eV) was used to calibrate the binding energy scale. CasaXPS software was used to analyze the recorded spectra. Background subtraction was performed using the Shirley function, and the product of Gaussian and Lorentzian functions was used for component fitting.

Out-of-plane GIWAXS measurements were performed with a Panalytical EMPYREAN X-ray diffractometer provided with a Co Kα X-ray source (λ = 1.78901 Å) at the anodic current of 50 mA and lamp voltage of 35 kV. Electrochemically modified ITO/PET electrodes were placed on a slightly disoriented monocrystalline silicon plate. The incident angle, on the order of 0.45°, was optimized for each sample. Measurements were carried out in the 31.5–68.5° 2θ range, with a step of 0.04°. The data were analyzed using the ICDD PDF-4+ database for the phase identification (Panalytical Inc., USA).

### Cytocompatibility Studies

The cytocompatibility of electrochemically modified ITO/PET electrodes was determined with a primary culture of a mixed neural population obtained from the mesencephalon of embryonic Sprague–Dawley rats and cultured for 3, 7, and 14 days, as described previously.^34,44^ Both unmodified ITO and a planar platinum electrode were used as control substrates. All experiments were performed in accordance with the EU guidelines (2010/63/UE) and were approved by the Health Products Regulatory Authority (AE19125/I179) and the local authority veterinary service. Every effort was made to minimize animal suffering and to reduce the number of animals used. An Olympus FluoView 1000 confocal microscope was used to visualize neuron and astrocyte cell populations marked through the indirect double-immunofluorescent labeling.^44,45^ The images were quantified to determine cell density and average neurite length, as reported previously.^45−47^ The biological experiments were conducted to include three biological replicates for all of the experimental groups. The results were expressed as the mean of the values ± the standard error of the mean. Comparisons among groups were performed by one-way ANOVA, followed by Bonferroni’s multiple comparison posthoc test. Statistical significance was considered at *p* < 0.05.

## Results and Discussion

### Electrochemical Modification

**Figure 1:**
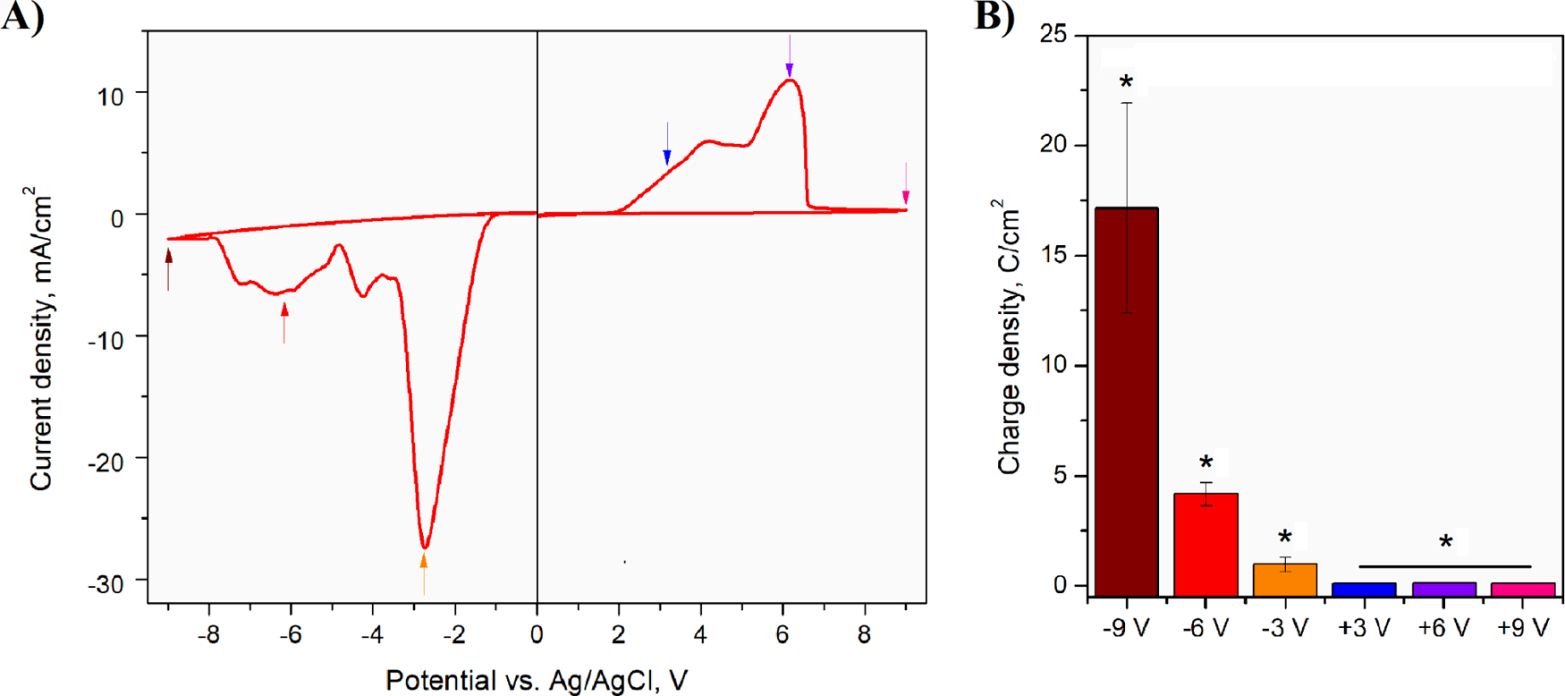
Cyclic voltammetric curves of ITO/PET substrates subjected to electrochemical oxidation (from 0 to 9 V, vs Ag/AgCl) and reduction (from 0 to −9 V, vs Ag/AgCl), collected in PBS supplemented with 0.1 M GluIL and 10 μM PSS, at a scan rate of 200 mV/s. Arrows indicate potentials chosen for subsequent electrochemical modification of ITO (A). Total charge density observed for ITO/PET when the electrode was subjected to electrochemical reduction and oxidation, * *p* < 0.05, *n* = 3 (B).

Electrochemical processing is acknowledged as an efficient method for the physicomechanical modification of metals and metal oxides by enhancing surface hardness, abrasion resistance, corrosion resistance, interfacial adhesion, and thermal and chemical stability.^48,49^ Due to the fact that sputter-deposited ITO film possesses a relatively smooth surface (*S*_a_ = 1.7 nm), an electrochemical process was employed to generate a nanostructured ITO topography for the promotion of cell adhesion.^50^ Cyclic voltammetry analysis (Figure 1A) has indicated the complex reduction/oxidation behavior of ITO within the potential range from −9 to 9 V (vs Ag/AgCl). Particularly, two peaks in the anodic region of the CV curve at +4 V and +6 V (vs Ag/AgCl) were attributed to the evolution of oxygen and anodic corrosion of ITO. This ITO corrosion was previously observed by Folcher et al.^51^ and was described as the breaking of In–O surface bonds by electrochemically generated radicals (OH^·^ and Cl^·^). Conversely, the cathodic region of the CV curve consisted of several reduction peaks at −3, −4, −6, and −7 V (vs Ag/AgCl). These peaks were related to the hydrogen evolution but also to the reduction of indium tin oxide and the formation of metallic tin and indium, as described previously.^52^

Cyclic voltammetry analysis was also performed to identify the optimal electric potential under which the electrochemical modification of ITO should be performed. Potential ranges of ±3 V (vs Ag/AgCl) were identified as the low potentials where the surface is supposed to be affected, ±6 V (vs Ag/AgCl) as the medium potentials where the main redox reactions are taking place, and ±9 V (vs Ag/AgCl) as the potentials where ITO is fully modified. The chronocoulometric curves indicating the increase in the cumulative charge density of ITO/PET electrodes when subjected to electrochemical reduction and oxidation can be found in the Supporting Information, Figure S1, and the total charge density of these processes is presented in Figure 1B. Interestingly, significantly higher charges were observed for ITO/PET electrodes subjected to electrochemical reduction than those subjected to electrochemical oxidation, and the highest charge was noticed for ITO/PET substrates subjected to −9 V (vs Ag/AgCl). The observed differences in the generated charge imply that reduction processes modified the ITO/PET surface chemistry to a greater extent than oxidation processes, which was further confirmed by the results of SEM/EDS, XRD, and XPS measurements.

### Chemical Composition

The chemical composition of unmodified and electrochemically modified ITO samples was investigated by XPS. Figure S2 presents a set of XPS spectra recorded for unmodified ITO. The survey spectrum (Figure S2A) revealed the presence of tin (Sn2p signal at ca. 490 eV), indium (In2p signal at ca. 450 eV), oxygen (O1s signal at ca. 530 eV), and carbon (C1s signal at ca. 285 eV).^53^ Notably, the presence of oxygen and carbon not only may be attributed to ITO and PET substrates, respectively, but also may be due to the so-called adventitious carbon.^53^ Analogous spectra were acquired for ITO samples electrochemically modified under various conditions.

**Figure 2:**
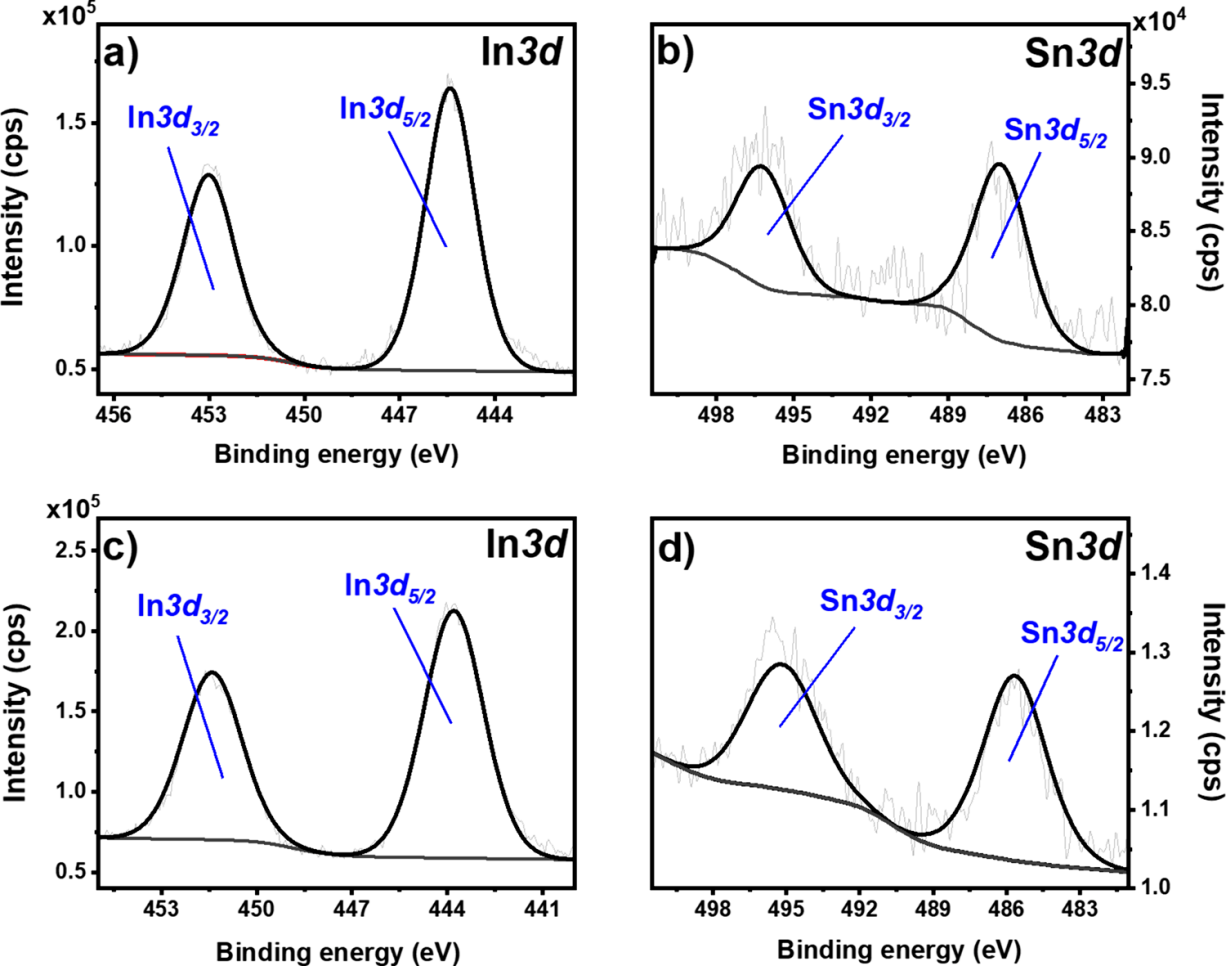
High-resolution XPS spectra of the (a,c) In*3d* region and (b,d) Sn*3d* region recorded for samples modified at +6 V (vs Ag/AgCl) (a,b) and −6 V (vs Ag/AgCl) (c,d).

High-resolution spectra of In3d and Sn3d characteristic regions were subsequently acquired to assess the chemical composition of the oxide layer. Figure 2 and Figure S2 present the high-resolution spectra of the In3d and Sn3d regions recorded for unmodified ITO and oxidized or reduced ITO, respectively. For both materials, one component with its spin–orbit splitting counterpart was present (In3d_5/2_ for the In3d region or Sn3d_5/2_ for the Sn3d region), indicating the chemical uniformity of the ITO surface. Electrochemical oxidation and reduction of ITO resulted in a shift in the position of the above-mentioned components (Table 1), which is a clear sign for a change in a chemical composition of ITO. For the unmodified ITO, indium was present in the form of In_2_O_3_ and tin in the form of SnO/SnO_*x*_. They were converted into In(OH)_3_ and SnO_2_ or elemental In and Sn under anodic or cathodic conditions, respectively.^52,53^ It was concluded that the formation of In(OH)_3_ resulted from a reaction of In with radical species formed during the anodization process, particularly OH^·^.^51^ Conversely, the deposition of metallic In and Sn was an obvious consequence of the electroreduction process.

**Table 1:** Summary of the Positions and Assignment of In*3d*_*5/2*_ and Sn*3d*_*5/2*_ Components and the In:Sn Ratio for Unmodified and Electrochemically Modified ITO

sample polarity (V)	position of In*3d*5/2 (eV)	assignment	position of Sn*3d*5/2 (eV)	assignment
+9	445.2	In(OH)_3_	487.0	SnO_2_
+6	445.4		487.0	
+3	444.6	In_2_O_3_	486.4	SnO/SnO_*x*_
0	444.5		486.6	
–3	444.1	In	485.6	Sn
–6	443.8		485.5	
–9	443.7		485.4	

### X-ray Diffraction Studies

**Figure 3:**
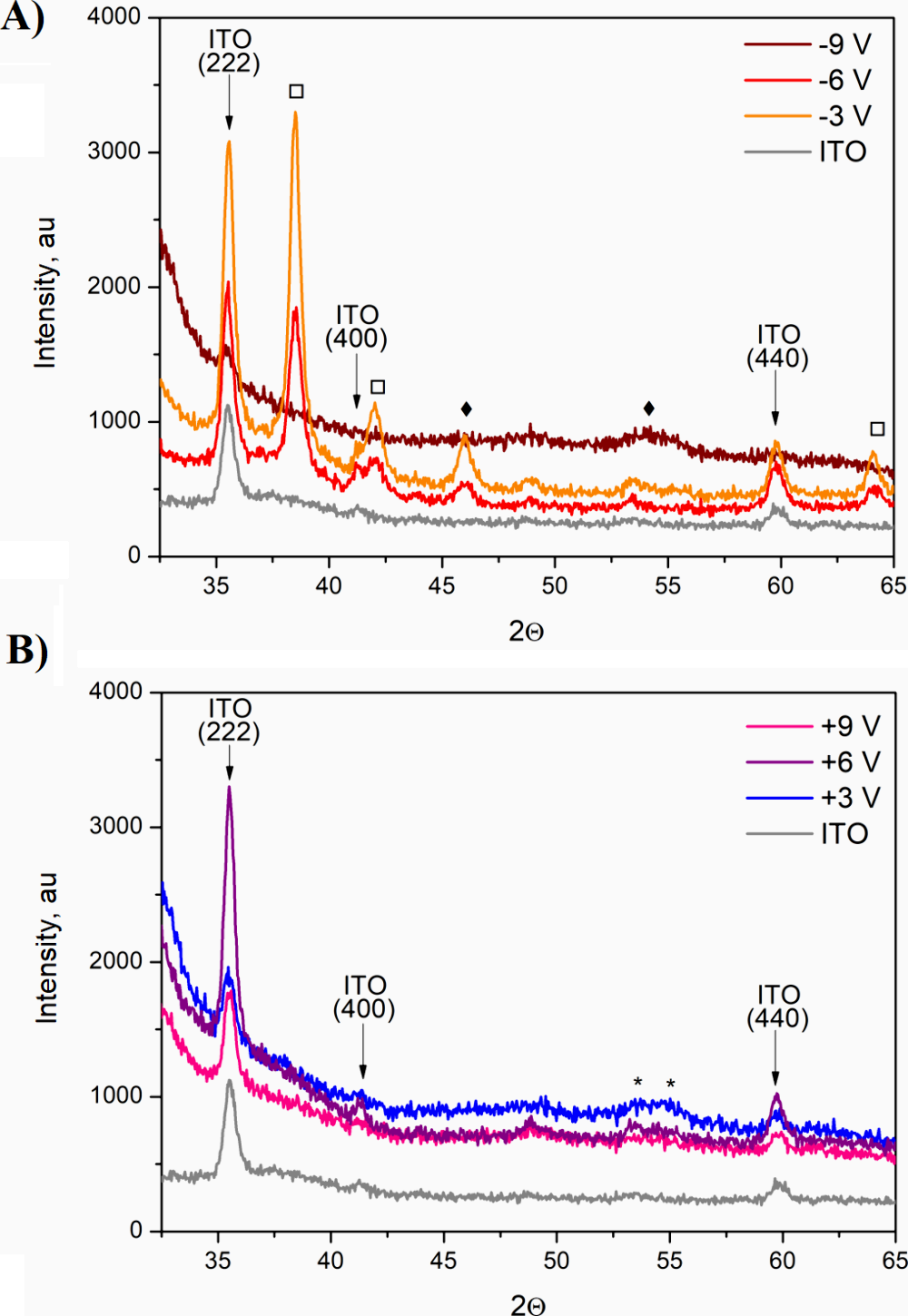
XRD patterns of electrochemically modified ITO/PET substrates subjected to reduction (A) and oxidation (B). ITO denotes cubic In_1.875_Sn_0.125_O_3_. A square denotes the metallic phase of indium: (101) at 38.50°, (002) at 42.02°, and (112) at 64.06°. A black diamond denotes the metallic phase of tin: (220) at 45.90° and (311) at 54.43°. An asterisk denotes In_4_Sn_3_O_12_.

Grazing incident XRD analysis (Figure 3) was employed to investigate structural changes in electrochemically modified ITO/PET substrates. The XRD pattern of an unmodified ITO consisted of a major peak of the (222) plane at 2θ of 35.54° and two less intensive peaks of the (400) and (440) planes at 41.26 and 59.66°, respectively, that correspond to cubic In_1.875_Sn_0.125_O_3_ (the main phase of ITO, ICDD card no. 01-089-4597, space group *Ia*3̅). With the application of an increasingly negative potential, the appearance of newly developed peaks at 38.50, 42.02, 45.90, 54.43, and 64.06° was observed, which were assigned to the metallic phases of indium (tetragonal, space group *I*4/*mmm*, ICDD card no. 00-005-0390) and tin (cubic, space group *Fd*3̅*m*, ICDD card no. 01-080-5354), and supporting the results of XPS analysis.^52^ The disappearance of a well-defined structure of the diffractogram was observed with ITO substrates reduced at −9 V (vs Ag/AgCl), indicating the loss of a crystalline structure. Conversely, the XRD diffractogram of samples subjected to oxidation potentials (+3 V) indicated the emergence of peaks at 53.38 and 54.65°, associated with the deposition of In_4_Sn_3_O_12_ (JCPDS card no. 85-084).^54−56^

### Optical Analysis

**Figure 4:**
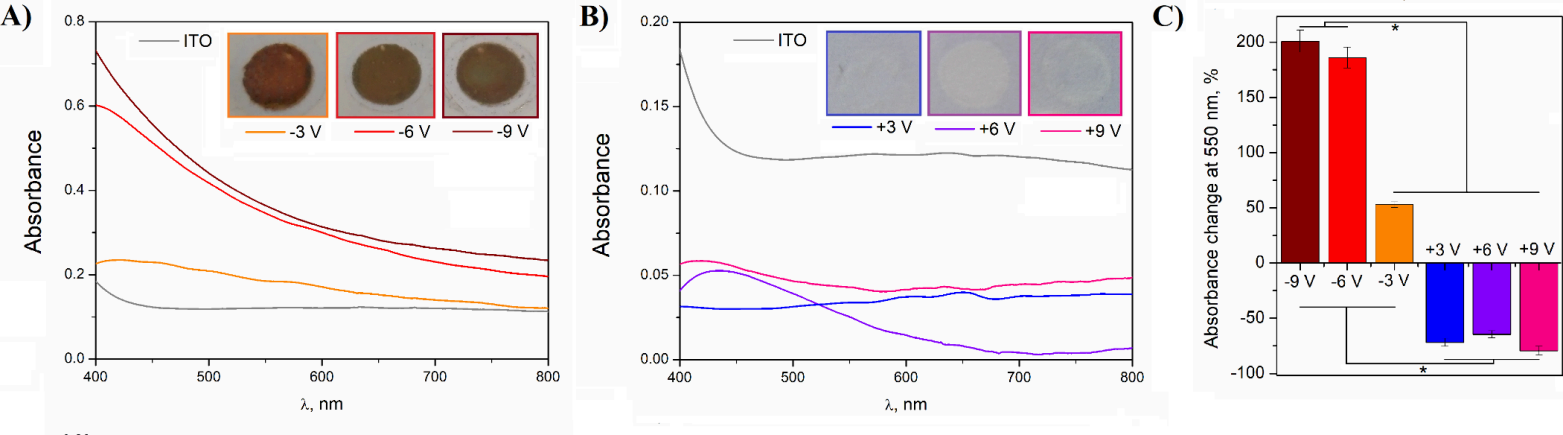
UV–vis spectra of reduced (A) and oxidized (B) ITO/PET substrates as well as macroscopic images of the electrochemically modified ITO electrodes (inset). The percentage change in absorbance of modified ITO/PET substrates subjected to electrochemical reduction and oxidation, relative to the absorbance of unmodified ITO/PET: * *p* < 0.05, *n* = 3 (C).

UV–vis spectra of electrochemically modified ITO/PET electrodes indicated a significant increase in the absorbance of the reduced substrates (Figure 4A), were easily observed as a discoloration on the ITO surface (Figure 4A inset), and were thought to be derived from the reduction of the oxides into metallic tin and indium. Conversely, the optical absorbance of all oxidized ITO substrates (Figure 4B) was significantly lower than that of the unmodified control ITO substrates. Specifically, an absorbance of 0.121 with a wavelength of 550 nm was observed with control ITO films and the application of positive potentials resulted in a 65% decrease in absorbance (for ITO/PET substrates oxidized at +6 V) and an 80% decrease in absorbance (for ITO/PET substrates oxidized at +9 V) (Figure 4C). Critically, it was shown that under specific oxidation conditions, it was possible to significantly increase the transparency of ITO in the visible spectrum.^57^

### Surface Morphology

**Figure 5:**
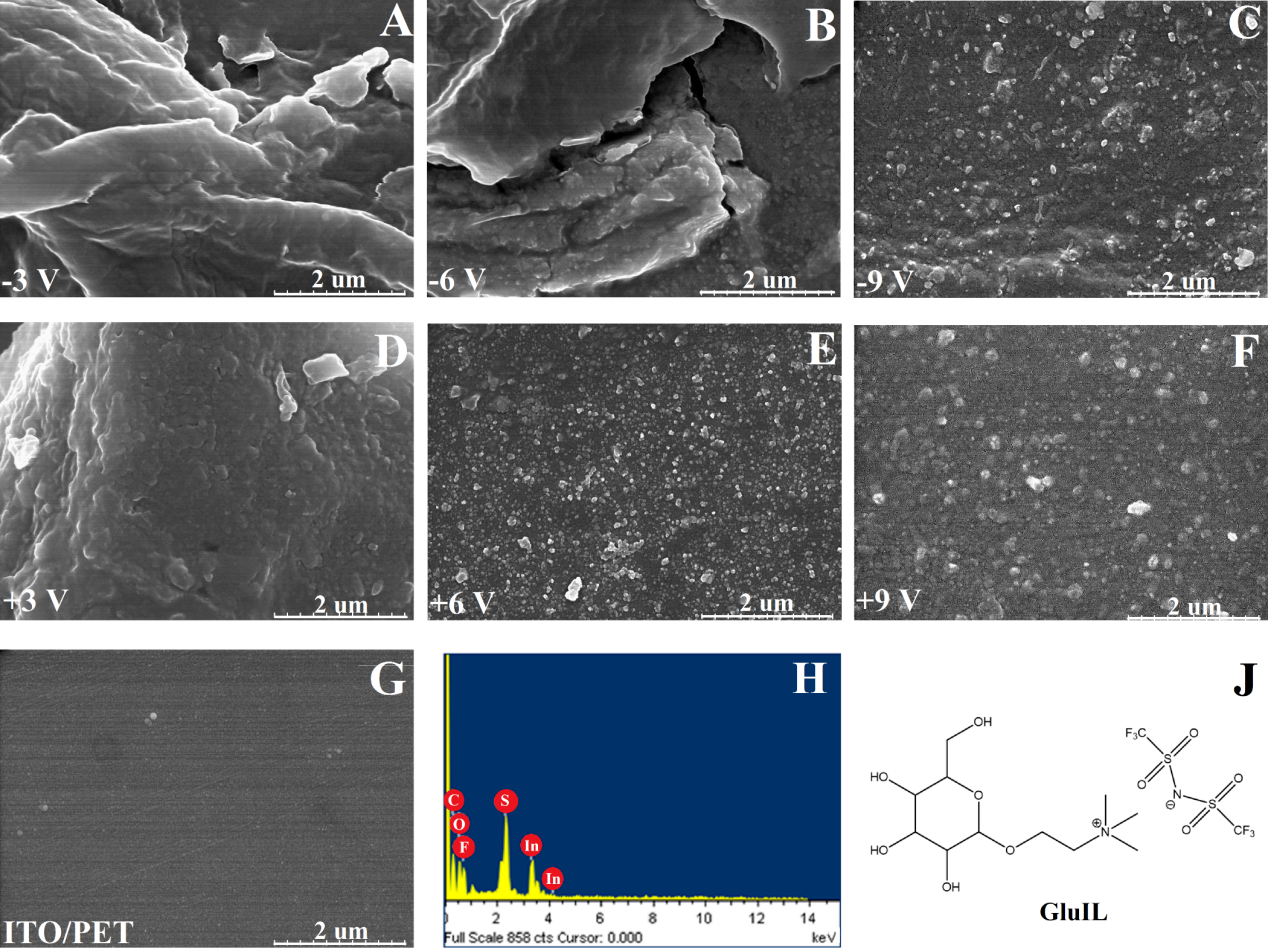
SEM images of ITO/PET electrochemically modified at −3 (A), −6 (B), −9 (C), + 3 (D), + 6 (E), and +9 V (F), as well as the unmodified ITO/PET substrate (G). The scale bar represents 2 μm. Representive results of EDS analysis for ITO/PET reduced at −9 V (H). The scheme of the chemical structure of GluIL (J).

Electrochemical reduction was observed to significantly affect surface morphology (Figure 5A–G) and roughness (Figure S3) of ITO/PET substrates relative to unmodified ITO (*S*_a_ of 1.7 nm), and the reduction with −3 and −6 V potentials caused the surface area to expand significantly and form out-of-plane undulations (*S*_a_ of 17 and 10 nm, respectively) on the ITO substrate, potentially offering advantages in the design of flexible and stretchable ITO devices.^58^ The complex morphology of ITO/PET substrates was significantly reduced with substrates treated at −9 V (*S*_a_ of 25 nm), possibly due to ITO degradation, as suggested by the XRD analysis. EDS analysis of ITO/PET substrates subjected to electrochemical reduction (Figure 5H) confirmed the presence of carbon (42.5 wt %), oxygen (28.1 wt %), fluorine (19.9 wt %), sulfur (6.2 wt %), and traces of indium (3.3 wt %), indicating the presence of the ionic liquid GluIL (sulfur and fluorine, Figure 5J). Conversely, ITO/PET substrates subjected to oxidation conditions exhibited a nanorough morphology, resulting from anodic corrosion,^51^ and the *S*_a_ value was observed to increase significantly from 17 to 78 nm (Figure S3).

### Electrochemical Behavior

**Figure 6:**
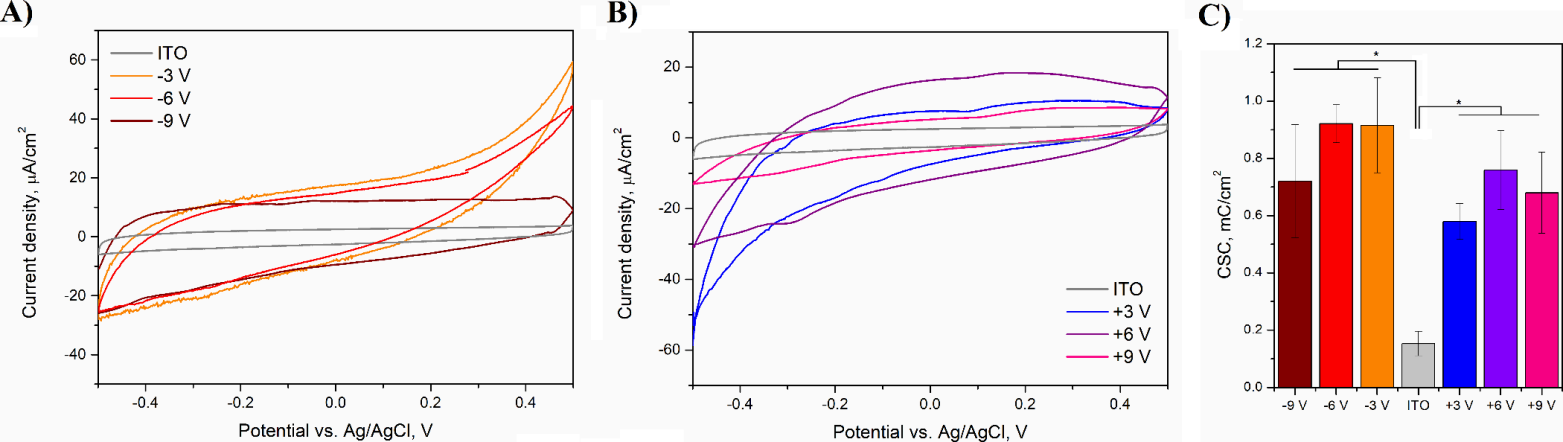
CV curves of reduced (A) and oxidized (B) ITO/PET electrodes conducted in PBS with a potential range from −0.5 to 0.5 V (vs Ag/AgCl) and a scan rate of 100 mV/s. CSC values of electrochemically modified ITO/PET calculated from corresponding CV curves (C). * *p* < 0.05, *n* = 3.

For the fabrication of effective recording or stimulation electrodes, electrochemically modified ITO/PET substrates should possess a relatively high CSC and low electrochemical impedance.^59^ When compared with a flat CV curve of unmodified ITO/PET, CV curves recorded for ITO/PET films subjected to electrochemical reduction (Figure 6A) and oxidation (Figure 6B) were more developed with larger anodic and cathodic currents. Also, the onset of either an anodic (at 0.5 V) or a cathodic (at −0.5 V) peak was observed in the CV curves of ITO/PET films subjected to electrochemical reduction and oxidation, respectively. Consequently, both reduction and oxidation processes led to an increase in the capacitance of ITO/PET substrates (Figure 6C), resulting in a 6-fold increase in CSC from 0.15 ± 0.04 mC/cm^2^ for unmodified ITO/PET to 0.92 ± 0.07 mC/cm^2^ for ITO reduced with a potential of −6 V. Basing on the shape of CV curves, which neither were ideally rectangular nor exhibited distinct redox systems, it could be stated that electrochemically modified ITO/PET films act as intercalation-type materials exhibiting some faradaic behavior.^60^ The increase in the capacitance should be, therefore, related to the increase in roughness^61^ (Figure S3) and chemical changes affecting their redox activity (summarized in the section describing the results of XPS studies), rather than the increase in the electroactive surface area (Figure S4).

### Impedance Analysis

**Figure 7:**
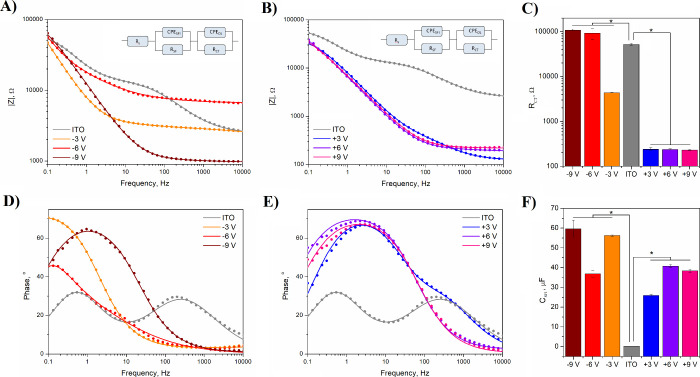
EIS analysis of electrochemically modified ITO/PET. Impedance vs frequency plots for ITO/PET substrates subjected to reduction (A) and oxidation (B). Symbols represent experimental data, and lines are the results of fitting of experimental data with the use of equivalent circuit model (inset). Surface resistance (*R*_SF_) of investigated ITO substrates (C). * *p* < 0.05, *n* = 3. Phase angle vs frequency plots for ITO/PET substrates subjected to reduction (D) and oxidation (E). Symbols represent experimental data, and lines are the results of fitting of experimental data with the use of equivalent circuit model. Solid/electrolyte interface capacitance (*C*_SEI_) of investigated surfaces (F). * *p* < 0.05, *n* = 3.

Impedance is a parameter describing the ability of a system to resist the flow of an alternating current; therefore, low impedance is an essential feature for any application requiring efficient charge transfer. A detailed description of the charge transfer process is possible through the analysis of the impedance behavior within a wide range of frequencies (encompassing the frequency range 50–300 Hz, relevant for neural stimulation^62,63^). EIS curves in the form of Bode plots extracted from nanostructured ITO/PET thin films (Figure 7A,B) have shown that ITO reduced and oxidized at all experimental potentials demonstrated a decrease in electrochemical impedance relative to a control, that is, unmodified ITO substrates. This has been thought to result from the increased roughness of modified surfaces, as an increase in the active electrode area will lower electrode impedance.^64^ An increase in impedance, however, has been observed with ITO substrates reduced to −6 V at frequencies above 100 Hz. It is noteworthy that this reduction condition also led to the lowest increase in roughness among all experimental groups (Figure S3).

To further study the electrochemical characteristics of electrochemically modified ITO/PET, equivalent electrical circuit modeling was employed. The model (Figure 7A,B insets) incorporated solution resistance (*R*_S_), which was connected in series with two RC elements, representing surface film resistance (*R*_SF_), solid/electrolyte interface capacitance (*P*_SFI_, *n*_SFI_), charge transfer resistance (*R*_CT_), and double-layer capacitance (*P*_DL_, *n*_DL_).^65,66^ The set of parameters resulting from the fitting procedure for EIS data acquired from electrochemically modified ITO/PET is presented in Table S1. Interestingly, the process of electrochemical oxidation was found to lead to a significant (2 orders of magnitude) decrease in the surface resistance (*R*_SF_) of ITO/PET relative to unmodified ITO/PET (Figure 7C). Conversely, electrochemical reduction of ITO/PET resulted in increased *R*_SF_ values, particularly with modification potentials of −6 and −9 V. As supported by XRD data, these modification potentials were shown to initiate ITO degradation processes, resulting in deteriorated electrochemical performance.

The phase angle versus frequency plots of electrochemically modified ITO/PET (Figure 7D,E) indicated a change in the capacitive behavior of all investigated surfaces. Unmodified ITO/PET exhibited two capacitive peaks at the frequencies ∼1 and ∼200 Hz, associated with solid/electrolyte interface capacitance and double-layer capacitance.^65,66^ The remodeling of the shape of phase plots occurred in response to electrochemical processing, and the formation of a single capacitive peak was observed. Interestingly, all oxidized ITO/PET surfaces exhibited a similar peak frequency at approximately 2 Hz. On the other hand, the position of a capacitive peak for ITO/PET reduced at −3, −6, and −9 V was 0.1, 0.1, and 1 Hz, respectively. With the aid of an equivalent circuit modeling of EIS data, it was possible to associate the changes in the phase angle with the increase in the solid/electrolyte interface capacitance (Figure 7F). As with CSC values, *C*_SEI_ was also found to significantly increase as the result of electrochemical processing of ITO/PET, changing from 0.18 ± 0.01 μF for unmodified ITO/PET to 59.6 ± 4.4 μF for ITO/PET subjected to −9 V. As the capacitance is strongly related to the surface morphology, it was believed that the observed changes in solid/electrolyte interface capacitance are the effects of the increase in roughness of ITO/PET as the result of electrochemical processing.^67^

### Transport Properties

Further electrical analysis of the effects of electrochemical modification of ITO/PET was assessed via four-point probe conductivity measurements from 4 to 300 K, providing data on the electronic transport as well as disorder of the surface layer (ITO/PET subjected to a potential of −9 V could not be assessed due to high resistance). As observed previously,^68^ unmodified ITO/PET presented a metal–insulator transition with decreasing temperature, exhibiting a transition temperature of ∼180 K and a room temperature conductivity of 205.6 S/cm. Minor changes in electrochemically modified ITO/PET suggested that the electrochemical treatment introduced disordered structure of the surface layer, the most notable for ITO/PET subjected to a potential of +9 V, which presented behavior typical for a semiconductor.^69^ Temperature dependence changes in conductivity (in particular, a decrease in conductivity at room temperature) were observed for all other electrochemically modified ITO/PET surfaces (Figure S5). The combined EIS analysis and four-point probe measurements indicate that the electrochemical processing may facilitate electron transport between the electrode and neural tissue, limiting charge transfer along the electrode. Previous studies^52^ indicated that the large separation between formed metallic particles is responsible for the significant deterioration in the surface conductivity of reduced ITO/PET and the surface conductivity is deteriorated in oxidized ITO/PET due to the anodic corrosion of ITO.^51^

### Cytocompatibility Studies

**Figure 8:**
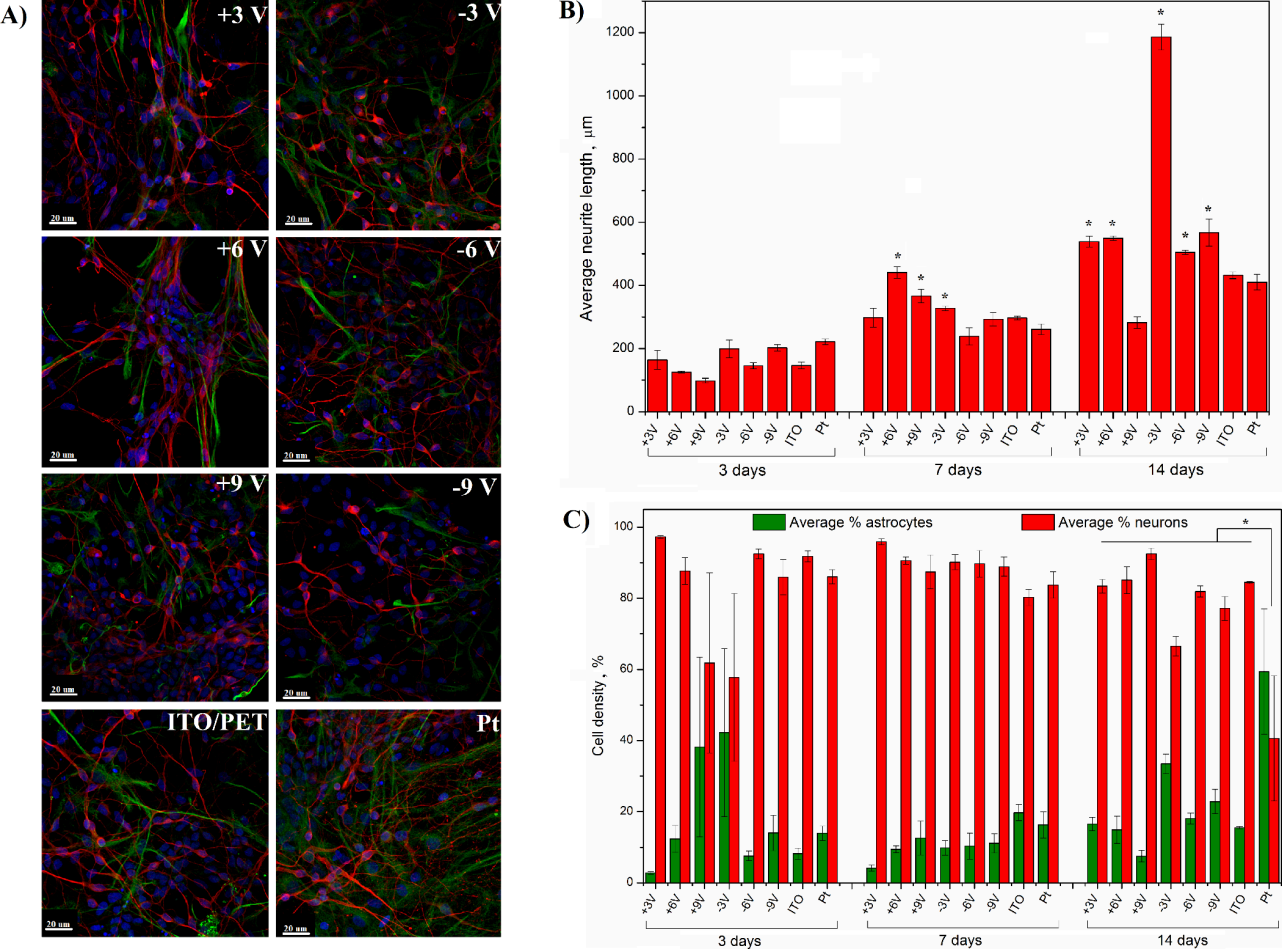
Fluorescent images of primary ventral mesencephalic mixed cell population cultured on reduced and oxidized ITO/PET as well as unmodified ITO/PET and Pt control substrates at 14 days (A). Neurons are visualized by anti-β III tubulin (red), astrocyte cells by anti-GFAP (green), and nuclei by DAPI (blue), scale bar = 20 μm. Cell density (%) of astrocytes and neurons on electrochemically modified ITO/PET, unmodified ITO/PET, and platinum control substrates (B) and average neurite length, μm (C). Results are expressed as the mean ± standard error of the mean, * *p* < 0.05, *n* = 3.

To verify whether electrochemical modification of ITO/PET through a GluIL electrolyte can affect the adhesion and growth of neural cells, a primary ventral mesencephalic mixed cell population was cultured for 3, 7, and 14 days on the surface of ITO/PET subjected to electrochemical oxidation and reduction, as well as on unmodified ITO/PET and a platinum control, the latter representing the current standard for neural electrode materials.^70^ At each time point, cells were fixed and immunostained with anti-β III tubulin for neurons, antiglial fibrillary acidic protein (GFAP) for astrocytes, and 4′,6-diamidino-2-phenylindole (DAPI) for cell nuclei, and the corresponding images (Figure 8A and Figure S6) were quantified to estimate the average neurite length (Figure 8B) and neuron-to-astrocyte ratio (Figure 8C).

The average neurite length was found to be similar on all investigated surfaces after 3 days in culture. By day 7, however, significantly longer neurites were observed on the surface of ITO/PET substrates modified with potentials of +6, + 9, and −3 V, relative to cells cultured on unmodified ITO/PET as well as the platinum control substrates. The effects of electrochemical modification were, however, the most significant after 14 days in culture, and the lengths of neurites of cells cultured on electrochemically modified ITO/PET substrates were significantly increased on all ITO substrates subjected to electrochemical modification except ITO substrates oxidized at 9 V (thought to be due to film instability^51^). Interestingly, a 2-fold increase in the neurite length in cells cultured on ITO/PET modified with a potential of −3 V was observed by day 14 relative to that in cells cultured on the control substrates. It is thought that the observed increase in neurite length was principally due to an increase in the roughness of ITO and possibly the presence of an ionic liquid moiety.

The influence of surface roughness on the attachment of neural cells has been already thoroughly investigated.^50,71,72^ It has been found that neurons do not readily attach on very smooth or rough surfaces, and the most favorable range of surface roughness for neural cells is between 50 and 70 nm.^50^ It is expected that topographic cues can also affect cell orientation and biocompatibility.^73^ The effect of surface roughness on cell adhesion is based on the possibility of establishing a contact area between a surface of the cell and a surface of a biomaterial, which is related to the interfacial adhesion force. Surface roughness in the optimum range (50–70 nm) may increase the contact area, resulting in an enhanced cell adhesion. The formation of focal adhesion, that is, protein structures forming mechanical links between cells and substrates, is a way to regulate cellular functions responsible for neurite outgrowth.^74^ The results of our preliminary studies with multiple ionic liquid chemistries (1-butyl-3-methylimidazolium tetrafluoroborate, 1-butyl-3-methylimidazolium trifluoromethanesulfonate, 1-ethyl-3-methylimidazolium octylsulfate, tetrabutylammonium lysine, tetrabutylammonium glutamine, 6-deoxy-6-trimethylammonio-d-glucose bistriflamide, (2-d-glucopyranosyloxyethyl)trimethylammonium bistriflamide, Figure S7) have shown that the average neurite length is highly dependent not only on the potential of modification but also on the chemical structure of the ionic liquid. In short, ionic liquids bearing the methylimidazolium-based cations either have hindered the extension of neuritis or have a weak positive impact of neural outgrowth. Ionic liquids composed of a tetrabutylammonium cation and either lysine or glutamine as an anion have a strong effect on neurite elongation, resulting in approximately 85% change in neurite outgrowth (Figure S7B), while the strongest effect on the extension of neurite length (approximately 105%) has been observed for an ionic liquid derived from (2-d-glucopyranosyloxyethyl)trimethylammonium cation and bistriflamide anion. Critically, it cannot be ruled out that GluIL residues act as an additional nutrient for cultured neurons, facilitating their growth and development.

Analysis of the neural-to-astrocyte ratio allowed further assessment of the in vitro cytocompatibility of electrochemically modified ITO^45^ and demonstrated a prevalence of neurons relative to astrocytes on all investigated materials at all three experimental time points. Conversely, platinum substrates facilitated the proliferation of astrocytes and reduced neural outgrowth, indicating that ITO/PET electrodes (unmodified or electrochemically processed) exhibited increased cytocompatiblitity relative to this control material. Consequently, the results of cytocompatibility studies further supported the hypothesis that electrochemically modified ITO/PET can serve as a neural interface and can be employed to substitute noble metals in neuroelectrode design.

Even though hybrid electro-optical stimulation is a novel concept, there were few studies that described its validation under in vitro and in vivo conditions, with the use of transparent and conducting electrodes. For instance, Zhang et al.^24^ used stretchable transparent electrodes developed by depositing carbon nanotube (CNT) thin film on PDMS and coating it with a photoresist (SU-8). The system was characterized with an impedance module of 0.2 MΩ at 1 kHz. A graphene-based, carbon-layered electrode array device, which was developed by Park et al.^75^ for neural imaging and optogenetic applications, was characterized with the impedance module of 400–700 kΩ at 1 kHz. Transparent and flexible low-noise graphene electrodes designed as suitable materials for both electrophysiology and neuroimaging exhibited the impedance module of 1 MΩ at 1 kHz.^76^ Electrochemically modified ITO films, particularly ITO/PET subjected to the oxidation process, exhibited an impedance module of only 0.2 kΩ at 1 kHz, greatly outperforming previously described materials.

## Conclusions

In this study, a new type of neural electrode material was successfully developed, namely, nanostructured ITO thin films electrochemically modified in the presence of a d-glucopyranoside-derived ionic liquid. Although the rate of the electrochemical modification was highest for ITO/PET subjected to electrochemical reduction, the oxidized ITO/PET samples were found to exhibit high transparency and low surface film resistance suitable for hybrid electrico-optical stimulation applications. ITO/PET substrates oxidized at +3 and +6 V were also observed to enhance neurite outgrowth, although this phenomenon was most significant in neurons cultured on ITO/PET substrates reduced at −3 V. Our results indicated that electrochemically modified, nanostructured ITO/PET is an advantageous material for the fabrication of flexible, transparent, conducting, and biocompatible neural interface, particularly suitable for the next generation of bio-optoelectronic applications.

## Supporting Information Available

Chronocoulometric curves of the processes of electrochemical modification of ITO (Figure S1), XPS survey spectrum and high-resolution spectra of In 3d and Sn 3d energy regions recorded for unmodified ITO/PET substrates (Figure S2), roughness of electrochemically modified ITO/PET (Figure S3), cyclic voltammograms of ITO/PET subjected to electrochemical reduction and oxidation recorded in the presence of a redox probe, K_4_[Fe(CN)_6_], relative electroactive surface area (Figure S4), conductivity as a function of temperature between 4 and 300 K (Figure S5), fluorescent images of primary ventral mesencephalic mixed cell population cultured on reduced and oxidized ITO/PET, as well as unmodified ITO/PET and Pt control substrates (Figure S6), fluorescent images of primary ventral mesencephalic mixed cell population cultured on ITO/PET subjected to electrochemical oxidation in the presence of various ionic liquids, the change in neurite outgrowth, and chemical structures of ionic liquids (Figure S7), the results of fitting procedure for EIS data (Table S1) (PDF)

## ABBREVIATIONS

CSC: charge storage capacity
CV: cyclic voltammetry
DAPI: 4′,6-diamidino-2-phenylindole
DBS: deep brain stimulation
EDS: energy-dispersive spectroscopy
EIS: electrochemical impedance spectroscopy
GFAP: glial fibrillary acidic protein
GluIL: (2-d-glucopyranosyloxyethyl)trimethylammonium bistriflamide
INS: infrared neural stimulation
ITO: indium tin oxide
PBS: phosphate-buffered saline
*P*_DL_: *n*_DL_, double-layer capacitance parameters
PET: poly(ethylene terephthalate)
*P*_SFI_, *n*_SFI_: solid/electrolyte interface capacitance parameters
PSS: sodium poly(styrenesulfonate)
*R*_CT_: charge transfer resistance
*R*_S_: solution resistance
*R*_SF_: surface film resistance
*S*_a_: surface roughness
SEM: scanning electron microscopy
XPS: X-ray photoelectron spectroscopy
XRD: X-ray diffraction
