# Supplementary material for: Flexible, Transparent, and Cytocompatible Nanostructured Indium Tin Oxide Thin Films for Bio-optoelectronic Applications
Source: ACS Appl Mater Interfaces. 2023 Sep 22;15(39):45701–12. doi: 10.1021/acsami.3c10861 (PMC10561142; doi:10.1021/acsami.3c10861)
Supplement: Supplementary file 1 — am3c10861_si_001.pdf [file am3c10861_si_001.pdf]

# Supporting Information

# Flexible, transparent and cytocompatible nanostructured indium tin oxide thin films for bio-optoelectronic applications

*Katarzyna Krukiewicz[1,2,*], Dominika Czerwińska-Główka[1], Roman Maria Turczyn[1,2], Agata Blacha-Grzechnik[1,2], Catalina Vallejo-Giraldo[3], Karol Erfurt[4], Anna Chrobok[4], Jérôme Faure-Vincent[5], Stéphanie Pouget[5], David Djurado[5], Manus J.P. Biggs[3,*]*

[1]Department of Physical Chemistry and Technology of Polymers, Silesian University of Technology, 44-100 Gliwice, Poland
[2]Centre for Organic and Nanohybrid Electronics, Silesian University of Technology, 44-100 Gliwice, Poland
[3]Centre for Research in Medical Devices, University of Galway, H91 TK33 Galway, Ireland
[4]Department of Chemical Organic Technology and Petrochemistry, Silesian University of Technology, 44-100 Gliwice, Poland
[5]CEA/INAC/SPrAM, Laboratoire d'Electronique Moléculaire Organique et Hybride, 38000 Grenoble, France

*corresponding authors: katarzyna.krukiewicz@polsl.pl, manus.biggs@universityofgalway.ie

A)

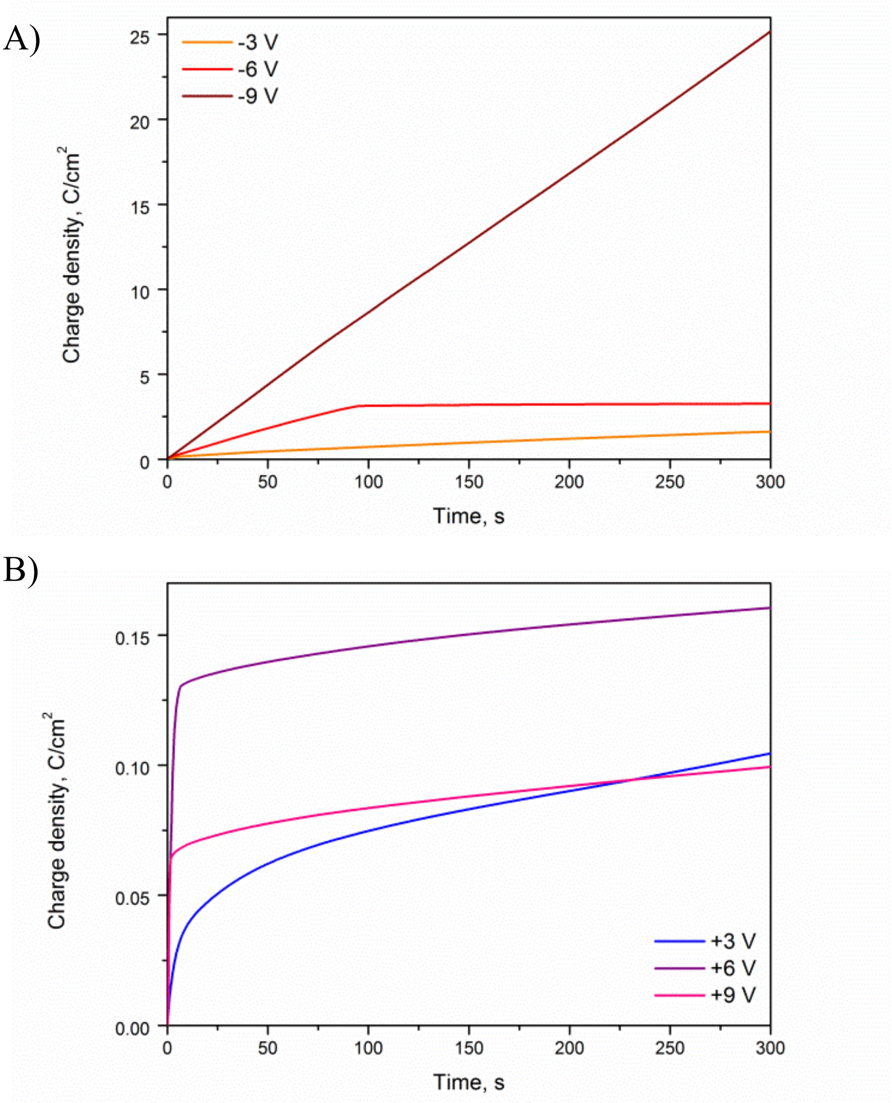


B)



Figure S1. Chronocoulometric curves showing the increase in the cumulative charge density of ITO/PET films when subjected reduction (A) and oxidation (B) in the electrolyte solution composed of PBS, 0.1 M GluIL and 10 μM PSS.

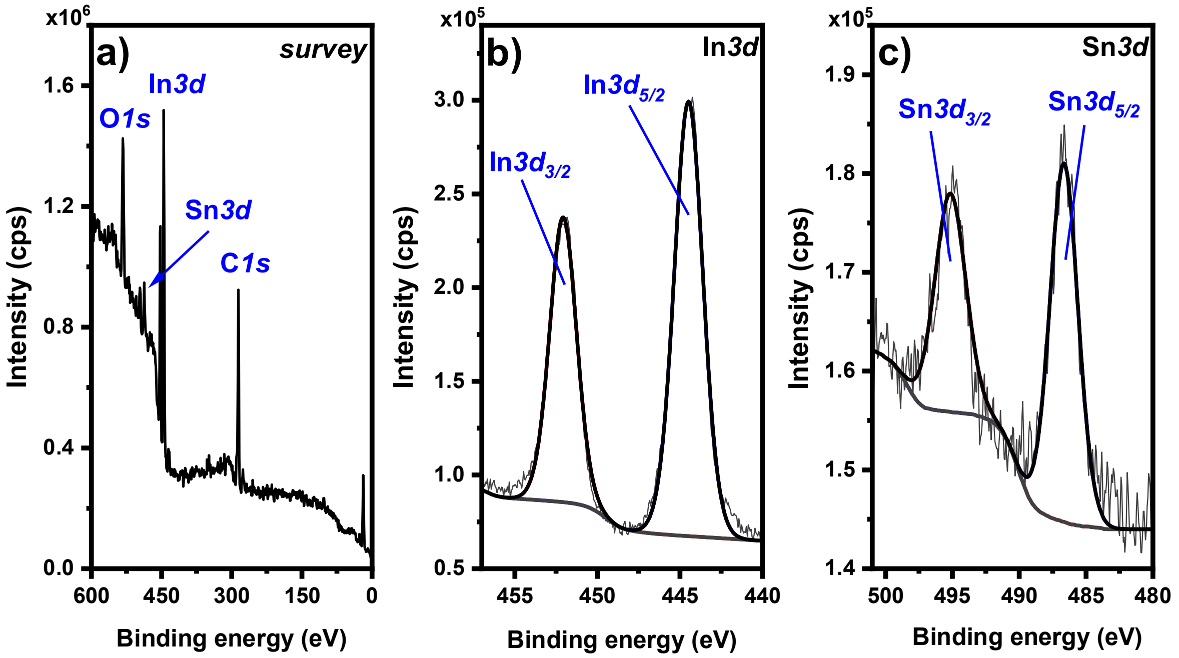


Figure S2. XPS survey spectrum (A) and high-resolution spectra of In3d (B) and Sn3d (C) energy regions recorded for unmodified ITO/PET substrates.

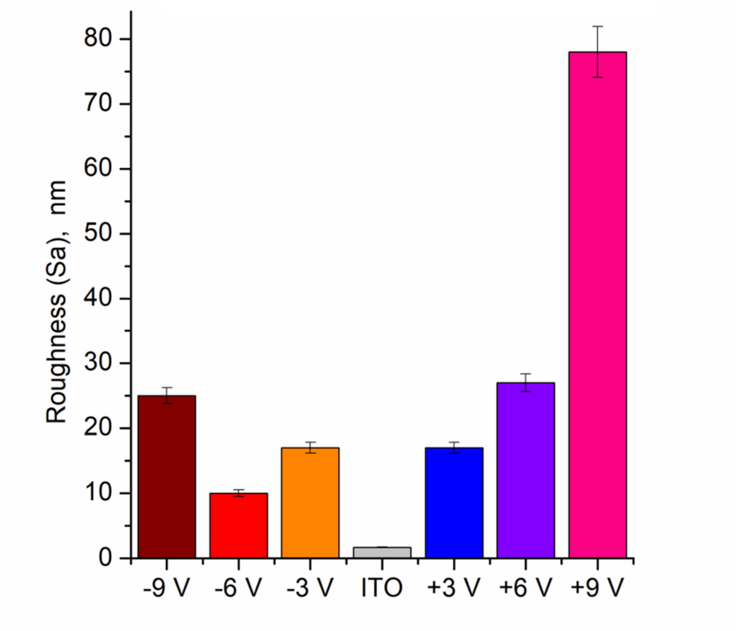


Figure S3. Roughness ($S_a$) of ITO/PET subjected to electrochemical reduction and oxidation.

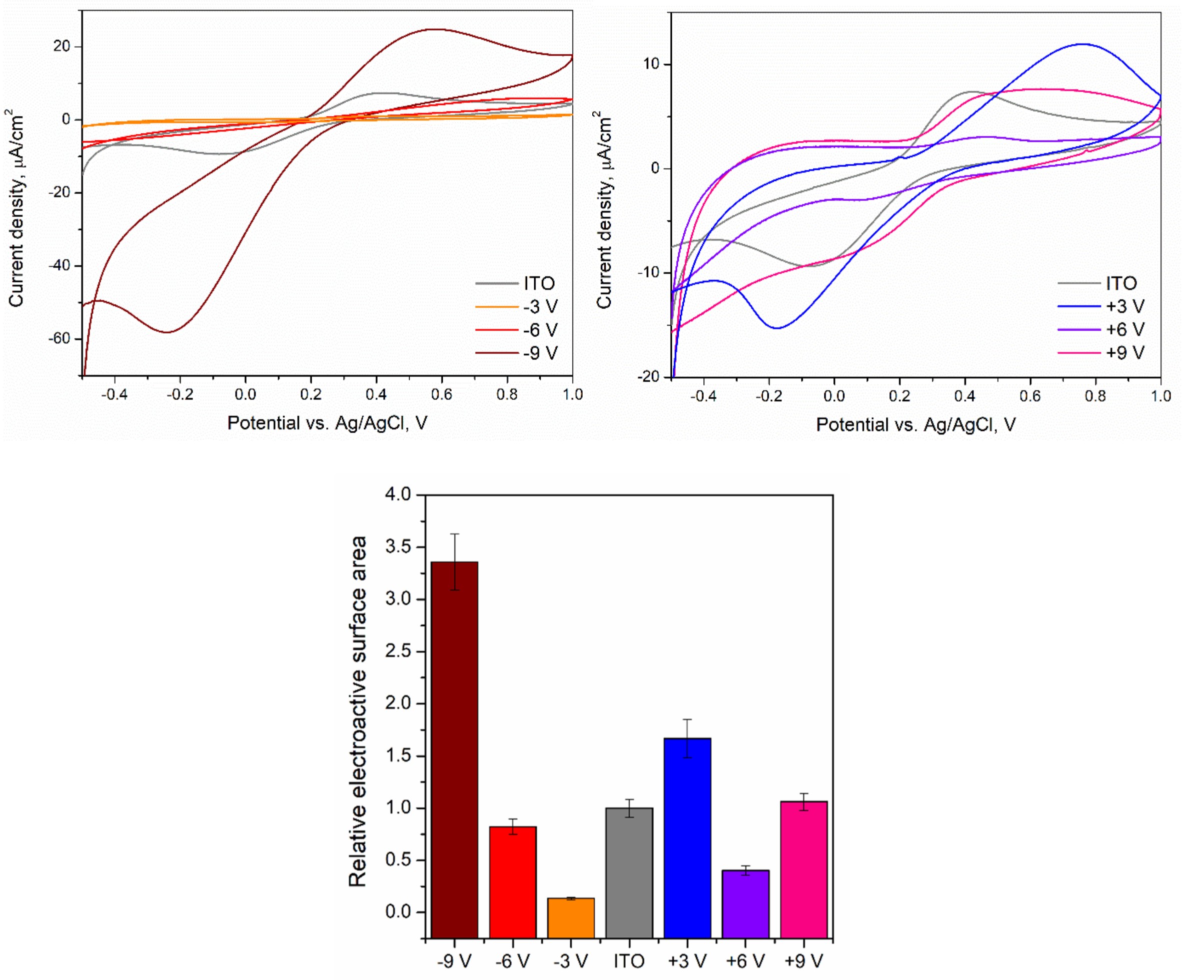


Figure S4. Cyclic voltammograms of ITO/PET subjected to electrochemical reduction and oxidation recorded in the presence of a redox probe, $K_4[Fe(CN)_6]$; relative electroactive surface area.

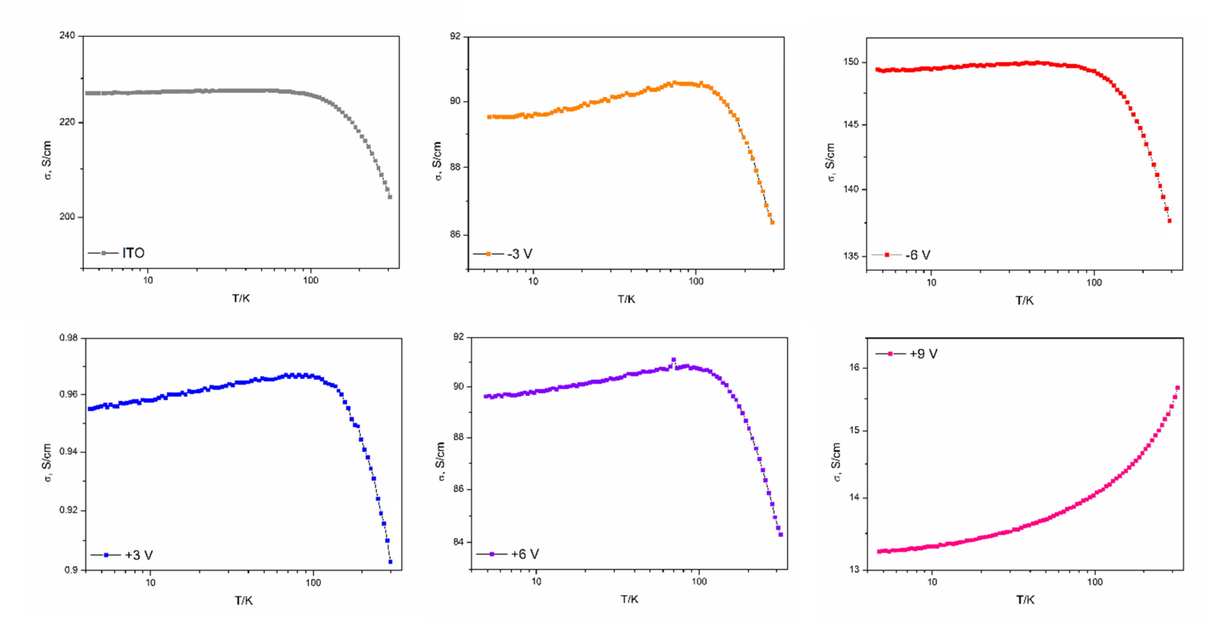


Figure S5. Conductivity as a function of temperature between 4 K and 300 K.

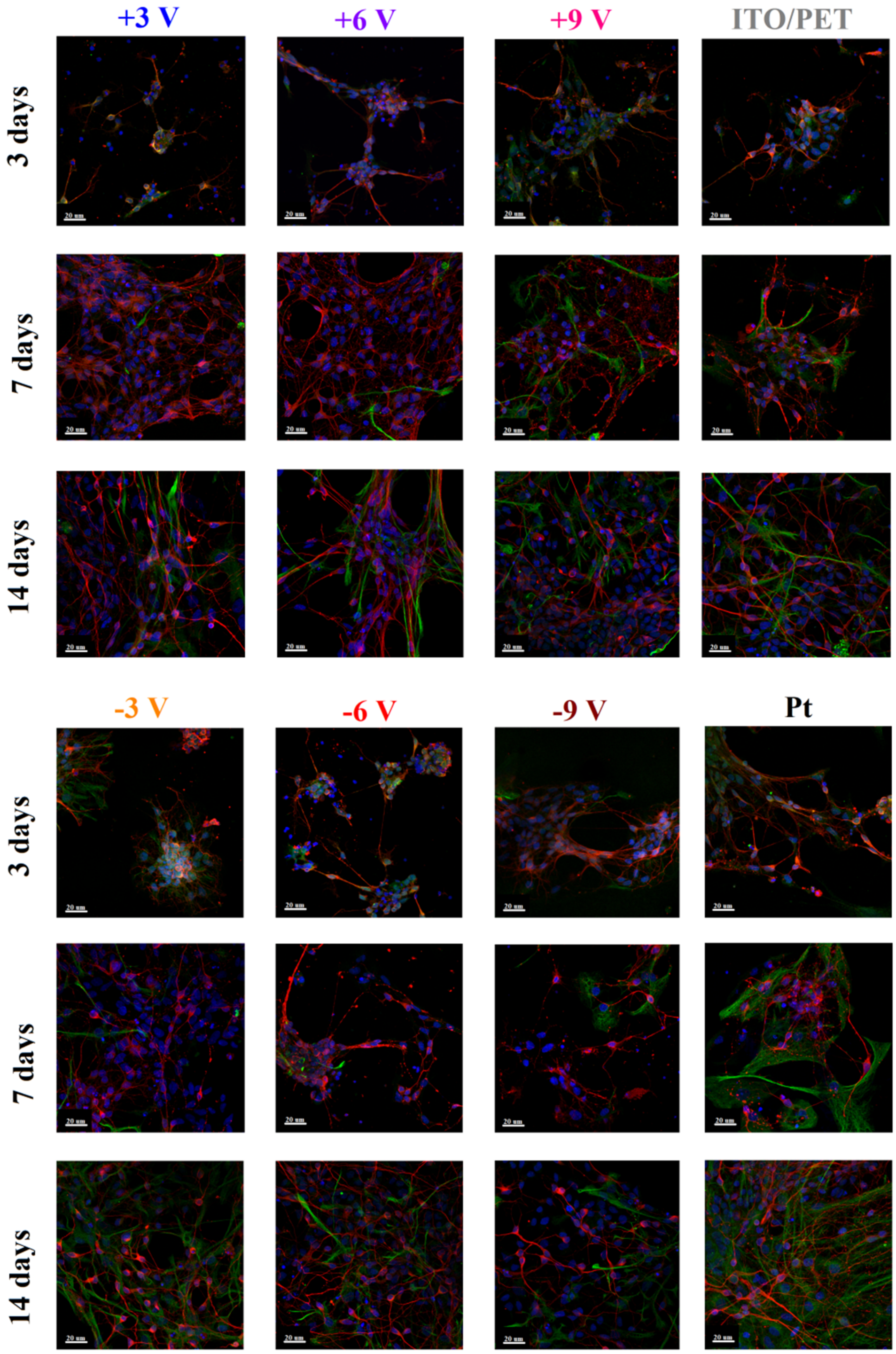


Figure S6. Fluorescent images of primary ventral mesencephalic mixed cell population cultured on reduced and oxidized ITO/PET, as well as unmodified ITO/PET and Pt control substrates for 3, 7 and 14 days; neurons are visualized by anti-β III tubulin (red), astrocyte cells by anti-GFAP (green) and nuclei by DAPI (blue), scale bar = 20 μm.

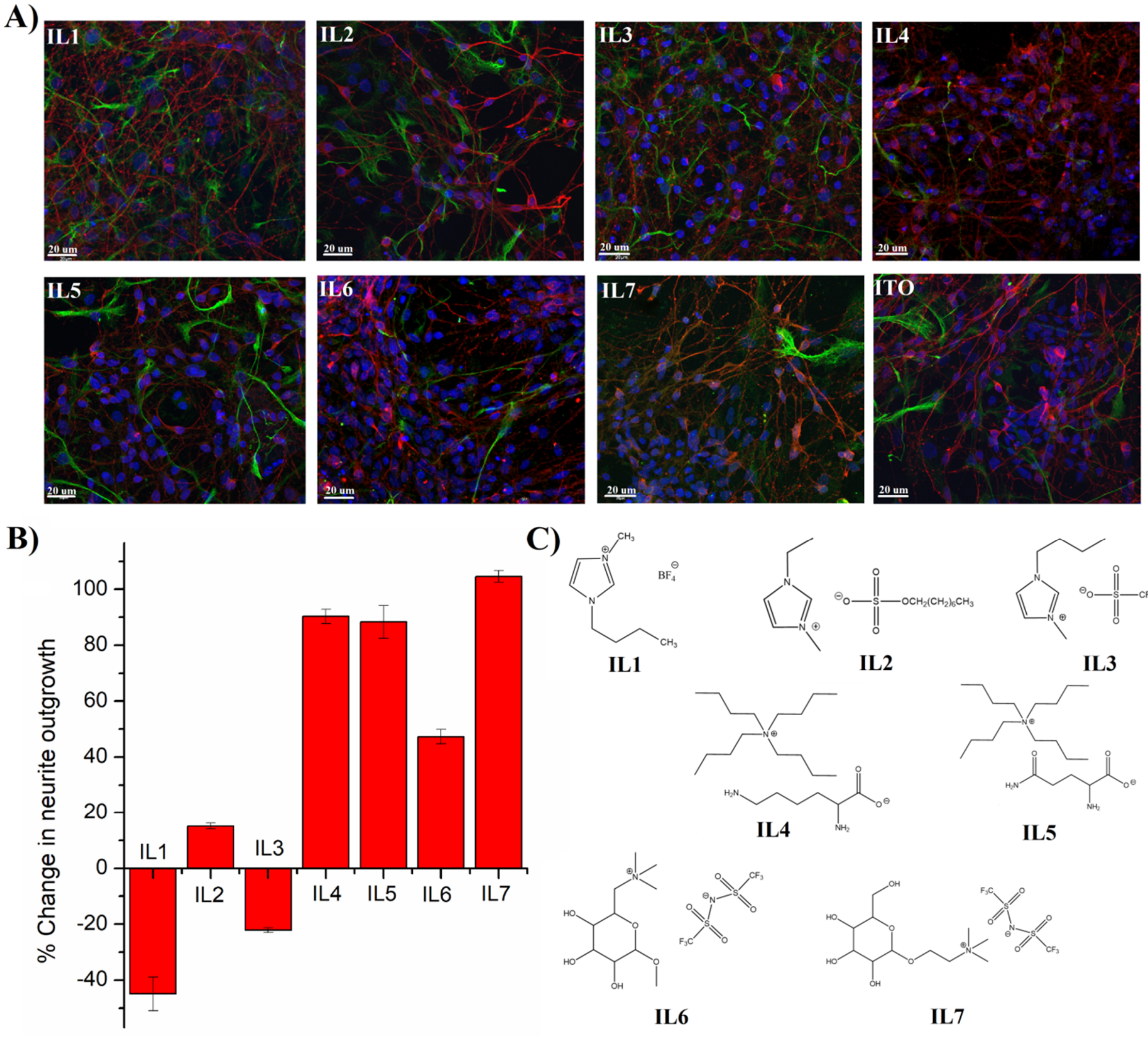


Figure S7. Fluorescent images of primary ventral mesencephalic mixed cell population cultured on ITO/PET subjected to electrochemical oxidation at +3 V (vs. Ag/AgCl) in the presence of various ionic liquids (0.1 M) dissolved in PBS solution containing 10 μM PSS, as well as unmodified ITO/PET for 7 days; neurons are visualized by anti-β III tubulin (red), astrocyte cells by anti-GFAP (green) and nuclei by DAPI (blue), scale bar = 20 μm (A). The change in neurite outgrowth, calculated as the percentage of the average neural length of cells cultured on electrochemically modifies ITO/PET relative to the average neural length of cells cultured on an unmodified control (B). Chemical structures of ionic liquids used in the process of electrochemical modification of ITO/PET(C).

Table S1. The results of fitting procedure for EIS data acquired from electrochemically-modified ITO/PET, as well as an unmodified ITO/PET control; $R_s$ – solution resistance, $R_{SF}$ – surface film resistance, $R_{CT}$ – charge transfer resistance, $P_{SFI}$ and $n_{SFI}$ – CPE parameters associated with solid/electrolyte interface, $P_{DL}$ and $n_{DL}$ – CPE parameters associated with double layer capacitance, $\chi^2$ – goodness of fit.

| | **-9 V** | **-6 V** | **-3 V** | **ITO** | **+3 V** | **+6 V** | **+9 V** |
|---|---|---|---|---|---|---|---|
| **$R_s$, Ω** | 985(±1%) | 6600(±2%) | 55(±18%) | 2426(±2%) | 123(±2%) | 18(±20%) | 20(±24%) |
| **$R_{SF}$, Ω** | 2590(±20%) | 36333(±13%) | 918550(±31%) | 11380(±2%) | 43353(±5%) | 90533(±12%) | 68416(±11%) |
| **$R_{CT}$, Ω** | 108280(±5%) | 92155(±28%) | 4347(±1%) | 51828(±5%) | 239(±8%) | 236(±3%) | 230(±3%) |
| **$P_{SFI}$** | 1.14E-04(±7%) | 3.16E-05(±5%) | 3.26E-05(±1%) | 1.28E-06(±3%) | 2.55E-05(±2%) | 3.28E-05(±2%) | 3.21E-05(±2%) |
| **$n_{SFI}$** | 0.653(±2%) | 0.479(±3%) | 0.862(±1%) | 0.684(±1%) | 0.85(±1%) | 0.833(±1%) | 0.815(±1%) |
| **$P_{DL}$** | 1.86E-05(±1%) | 4.13E-05(±6%) | 5.45E-05(±1%) | 1.63E-05(±3%) | 3.20E-05(±7%) | 5.00E-04(±9%) | 5.00E-04(±19%) |
| **$n_{DL}$** | 0.874(±1%) | 1(±3%) | 0.098(±1%) | 0.824(±2%) | 0.708(±1%) | 0.099(±10%) | 0.027(±73%) |
| $\chi^2$ | 2.93E-04 | 8.68E-04 | 4.60E-05 | 3.94E-04 | 7.12E-04 | 7.83E-04 | 1.12E-03 |
